# Determining the molecular landscape and impact on prognosis in HPV-associated head and neck cancer

**DOI:** 10.1186/s41199-020-00058-2

**Published:** 2020-09-09

**Authors:** Suchin Khanna, Sarah Palackdharry, Logan Roof, Christina A. Wicker, Jonathan Mark, Zheng Zhu, Roman Jandorav, Alfredo Molinolo, Vinita Takiar, Trisha M. Wise-Draper

**Affiliations:** 1 Division of Hematology/Oncology, Department of Internal Medicine, Yale University School of Medicine, 333 Cedar Street WWW 201 Attn: Suchin Khanna, New Haven, CT 06510 USA; 2 Division of Hematology/Oncology, Department of Internal Medicine, University of Cincinnati College of Medicine, Cincinnati, OH USA; 3 Department of Radiation Oncology, University of Cincinnati College of Medicine, Cincinnati, OH USA; 4 Department of Otolaryngology, Eastern Virginia Medical Center, Norfolk, VA USA; 5 Department of Environmental Health, University of Cincinnati College of Medicine, Cincinnati, OH USA; 6 Department of Pathology, UC San Diego Moores Cancer Center, La Jolla, CA USA

**Keywords:** Head and neck, Oropharynx, Molecular biology, Human papillomavirus, pAMPK

## Abstract

**Background:**

Human papillomavirus (HPV) associated head and neck squamous cell carcinoma (HNSCC) has a better prognosis than HNSCC due to other risk factors. However, there is significant heterogeneity within HPV-associated HNSCC and 25% of these patients still do poorly despite receiving aggressive therapy. We currently have no good molecular tools to differentiate and exclude this “high-risk” sub-population and focus on “low-risk” patients for clinical trials. This has been a potential barrier to identifying successful de-escalation treatment strategies in HPV-associated HNSCC. We conducted an analysis of molecular markers with a well-known role in the pathogenesis of HPV-associated HNSCC and hypothesized that these markers could help independently predict recurrence and prognosis in these patients and therefore help identify at the molecular level “low-risk” patients suitable for de-escalation trials.

**Methods:**

We analyzed 24 tumor specimens of patients with p16+ HNSCC who underwent definitive resection as primary treatment. Tissue microarray (TMA) was generated from the 24 pathology blocks and immunohistochemistry (IHC) was performed using highly specific antibodies for our chosen biomarkers (PI3K-PTEN, AKT pathway, mTOR, 4EBP1, S6, and pAMPK, ERCC-1). Transcriptome data was also obtained for 7 p16+ HNSCC patients from The Cancer Genome Atlas (TCGA). Data from the TMA and TCGA were analyzed for association of relapse-free survival (RFS) and overall survival (OS) with protein and gene expression of the chosen biomarkers.

**Results:**

Increased pAMPK protein activity by IHC and AMPK gene expression by TCGA gene expression data was correlated with improved RFS with a trend towards statistical significance.

**Conclusions:**

This data suggests that increased pAMPK activity and expression may portend a better prognosis in HPV-associated HNSCC undergoing primary definitive resection. However, these findings require validation in larger studies.

## Background

Head and neck squamous cell carcinoma (HNSCC) is the sixth most common cancer type worldwide, accounting for more than 550,000 cases and 380,000 deaths annually [1]. Human papillomavirus (HPV) is a significant risk factor and is particularly prevalent in oropharyngeal squamous cell carcinomas (OPSCC). The incidence of OPSCC has been on the rise over the past three decades, with multiple lines of evidence linking the rise of OPSCC incidence to the rise in HPV [2, 3]. HPV-associated OPSCC is thought to be a distinct clinical and molecular entity with unique histopathological features as compared to tobacco and alcohol related HNSCC [4]. HPV-associated HNSCC has a better prognosis with improved survival and enhanced response to treatment, irrespective of treatment modality, compared to HNSCC due to other risk factors [5–7].

Despite the different epidemiology, natural history and treatment response, HPV-associated OPSCC is currently managed in the same way as HPV-negative cancer, often with toxic multimodality therapy [8]. Patients can experience significant morbidity and diminished quality of life.

Numerous recent clinical trials have studied de-escalation treatment strategies in HPV-associated OPSCC in the hope of reducing toxicities while maintaining high cure rates, however none of these trials have been practice changing. In a multicenter cooperative group study, ECOG 1308, 80 patients with Stage III or IVA (using AJCC 7th Edition Staging Manual) HPV-associated OPSCC received induction chemotherapy with three cycles of cisplatin, paclitaxel, and cetuximab followed by cetuximab combined with a lower dose of radiation therapy (RT), 54 Gy in 27 fractions, for those patients who achieved a clinical complete response to induction chemotherapy at the primary site. With the lower dose of radiation, the two-year progression-free survival (PFS) was 80% and two-year overall survival (OS) was 94% [9]. These results are similar to those of the ECOG 2399 study, the Lancet Oncology study by Chen et al., and the OPTIMA study [10–12]. However, much like ECOG 1308 all of these studies included a small number of patients and therefore no definitive conclusion about this de-escalation strategy can be made without further data from larger studies.

A more recent and large randomized study, RTOG 1016 which studied concurrent chemoradiation with cetuximab versus cisplatin in HPV-associated OPSCC reported worse overall survival and progression-free survival for the hypothesized less toxic concurrent cetuximab/RT arm compared to cisplatin/RT and surprisingly and importantly also showed similar toxicity [13]. A similar trial comparing cetuximab/RT versus cisplatin/RT, TROG 12.01, is ongoing as are several other de-escalation trials [14–16] and it remains to be seen whether any of these trials will be practice changing. The DE-ESCALATE HPV trial, like RTOG 1016, also compared effectiveness and toxicity of cisplatin/RT versus cetuximab/RT in HPV-associated OPSCC. It showed consistent findings with RTOG 1016, namely that there was no benefit in terms of reduced toxicity with cetuximab but there was inferior tumor control and survival outcomes with cetuximab [17].

A potential barrier that RTOG 1016, DE-ESCALATE HPV, and other similar trials are experiencing is that HPV-associated OPSCC is a heterogeneous disease with up to 25% of these patients still doing poorly despite receiving aggressive therapy. These 25% of patients are therefore unsuitable candidates for de-escalation strategies, however we currently have no way of identifying this sub-population to exclude them from trials. It is therefore possible that RTOG 1016 and other de-escalation studies have failed to maintain equivalent outcomes because these trials do not optimally risk-stratify HPV-positive patients. With improved means of identifying characteristics of the subset of HPV-associated OPSCC patients with better prognoses we might be able to better identify suitable candidates for de-escalation strategies in future trials.

The molecular basis of the difference in outcomes amongst the HPV-associated OPSCC subgroups remain elusive. Finding such molecular means of prognosticating and risk-stratifying HPV-associated OPSCC patients will be profoundly useful in the future to more accurately select suitable candidates for de-escalation treatment strategies as well as to determine appropriate primary modality of treatment. We conducted an analysis of molecular markers with these goals in mind. We specifically analyzed HPV-associated OPSCC patients that underwent definitive resection as their primary modality of treatment. We studied biomarkers with a well-known role in the molecular pathogenesis of HPV-associated OPSCC with the hypothesis that these markers could help predict recurrence and prognosis in these patients after surgery. These markers included proteins in the PI3K-PTEN-AKT pathway, mTOR, 4EBP1, S6, and pAMPK, ERCC-1 [18–22].

## Methods

### Study participants

This is an IRB-approved (University of Cincinnati IRB#: 2016–0532) retrospective study that analyzed the tumor specimens of patients with p16+ OPSCC by immunohistochemistry who underwent definitive resection as primary treatment from May 2010 to April 2016. Inclusion criteria included adult patients with HPV-associated OPSCC that underwent definitive surgical resection and are followed at the University of Cincinnati. Exclusion criteria included patients with incomplete records, patients with prior history of HNSCC, patients with multiple simultaneous cancers, pregnant women, children (age < 18), and prisoners. The following characteristics were obtained and accounted for in each patient: Age, Gender, Location of Tumor, TNM Staging of Tumor at time of definitive resection, Date of Definitive Resection, Date of Last Follow-Up, Recurrence Status, Date of Recurrence (if applicable), Date of Death (if applicable), Adjuvant Treatment (if any), Surgical Margin status, Lymphovascular Invasion (LVI), Perineural Invasion (PNI), Extracapsular Extension (ECE), Overall Survival (OS), and Relapse-Free Survival (RFS). Twenty-six resected tumor specimens from patients that met the criteria highlighted above were screened, but only 24 patients had sufficiently high-quality tissue staining for analysis.

### Molecular markers

We studied biomarkers with a well-known role in the molecular pathogenesis of HPV-associated OPSCC One of the most frequently altered pathways in cancer with mutations in up to 30% of HNSCC is the PI3K pathway which regulates physiologic cellular processes including cell proliferation, differentiation, motility, metabolism, and apoptosis. Its central components, PI3K, AKT, and mTOR, drive tumor metastasis by promoting cell motility. PTEN acts as a negative regulator of this pathway. Downstream effectors of the PI3K pathway include pS6 and 4EBP1 [19, 23]. Head and neck tumors often increase glycolysis to generate ATP, even in the presence of normal oxygen concentrations since this is much faster than oxidative phorphorylation in order to meet the tumor’s increased energy and biosynthesis needs. Aerobic glycolysis, as this process is called, is regulated by aberrant signaling pathways including the AMPK pathway [24]. ERCC-1 has been theorized to have a role in prognosis and response to treatment in HNSCC, and this biomarker was therefore also included in this study [22].

### Tissue microarray, immunohistochemistry

A tissue microarray (TMA) was generated from the 24 pathology blocks with 1 mm core size. Tumor specimens were de-identified and sent for molecular analysis using immunohistochemistry (IHC). The collected tissues were dehydrated and embedded in paraffin following standard procedures. Hematoxylin-eosin stained 5 μm sections were used. Unstained sections were deparaffinized and processed for IHC. IHC was performed using highly specific antibodies for our chosen biomarkers (Table 1). The antibodies used and the procedures carried out involved overnight incubation with the primary antibody, biotinylated secondary antibodies, and the ABC method to detect the presence of reactive antibodies recognizing each protein. Our pathologist who was blinded to any clinical information independently analyzed the IHC-stained biopsy slides. For every case, the whole tumor areas were quantified. The number of positive cells were visually evaluated for each tissue and the results were re-expressed as a percentage of stained cells out of the total number of cells. The intensity of immunoreactivity was also evaluated with scores of 1 for weak, 2 for moderate, and 3 for strong. An H-score was then calculated by multiplying both parameters [25].

**Table 1:** List of antibodies for immunohistochemistry of tumor samples

Antibody Targets	
Phospho-AKT (Ser473)	
Phospho-4E-BP1 (Thr37/46) (236B4)	
Phospho-S6 Ribosomal Protein (Ser235/236)	
ERCC1 (D6G6)	
mTOR (7C10)	
PTEN (138G6)	
Phospho-mTOR (Ser2448) (49F9)	
Phospho-AMPKα (Thr172) (40H9)	
PI3 Kinase p110α (C73F8)	
Cyclin D1 (92G2)	
Phospho-AKT (Thr308) (244F9)	

### The Cancer Genome Atlas

Transcriptome data (mRNASeq2, level 3) was obtained from The Cancer Genome Atlas (TCGA). Data were analyzed for RFS with regards to gene expression of AMPK (PRKAA1), PI3K (PIK3CA), AKT1, AKT2, AKT3, ERCC1, S6 (RPS6), PTEN, mTOR, EIF4EP1 (4E-BP1).

### Statistical analysis for protein activity and gene expression analysis

For protein expression analysis, data were analyzed using the Cox Proportional Hazards model for the primary outcomes, relapse-free survival (RFS) and overall survival (OS). RFS was defined as the duration of time from surgery to time of relapse and OS was defined as the duration of time from diagnosis until time of death. All hazard ratios and/or odds ratios and 95% confidence intervals were reported. Kaplan-Meier curves were created, and comparisons of Kaplan-Meier survival data between different subgroups and the corresponding hazard functions were conducted by two-sided log-rank tests. *P*-values less than 0.05 were reported as indicators of significant difference between the subgroups. All analyses were conducted using R 3.1.3.

For gene expression analysis, Kaplan-Meier curves were created, and RFS was compared between individuals with mRNA levels ≥ the median and < the median values for each given target. Log-rank *P*-values of less than 0.05 were reported as indicators of significant difference between the subgroups. Statistical analyses were conducted using R.3.6.3 “Holding the Windsock.”

## Results

### Patient characteristics

For immunohistochemistry analysis, tumor tissue specimens from 26 patients that met eligibility criteria for this retrospective study were identified through the head and neck cancer tissue database in University of Cincinnati’s Department of Pathology. Patient characteristics are summarized in Table 2A. The median age was 61.5, with a range from 39 to 83 years of age. The majority of patients were male (89%). About half of the patients studied were found to be Stage I at diagnosis (46%) by the AJCC Eighth Edition Staging Manual, half of the patients were found to be Stage II (50%), and 1 of the 26 (4%) patients was Stage III. Regarding high risk features, 8 of the 26 patients (31%) were found to have positive surgical margins and 6 of the 26 patients (23%) were found to have extracapsular extension (ECE) after surgical resection. A total of 12 patients (46%) had at least one high-risk feature (either ECE or positive surgical margins). Many of the patients were also noted to have intermediate risk features such as lymphovascular invasion (31%), perineural invasion (12%), T3/T4 disease (15%), or N2/N3 disease (46%). Overall, 5 patients (19%) had intermediate risk disease. Median follow-up was 18.3 months. Fourteen percent of the patients had recurred and 11% had died at the time the retrospective study was conducted. Approximately 40% of patients received adjuvant treatment after definitive surgery, specifically those with high-risk (adjuvant concurrent chemoradiation) and intermediate-risk (adjuvant radiation therapy only) disease.

**Table 2:**
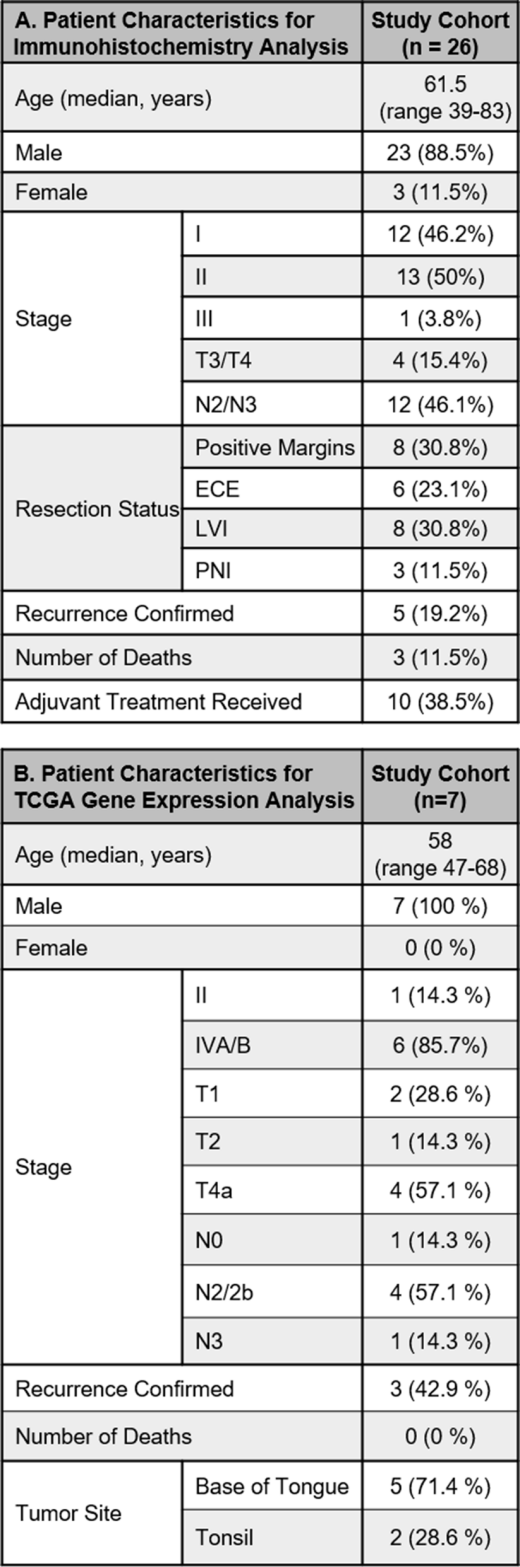
Characteristics of patients in the study sample. A) Patient characteristics for immunohistochemistry analysis. B) Patient characteristics for TCGA gene expression analysis

For gene expression analysis, transcriptome data was obtained from The Cancer Genome Atlas (TCGA). Patient characteristics are summarized in Table 2B. There were 7 p16+ patients with recurrence data. The median age was 58, with a range from 47 to 68 years of age. All of the patients were male. Staging data was determined by the AJCC Seventh Edition Staging Manual. One (14.3%) patient was Stage I, 6 (85.7%) were stage IVA/B. Two (14.3%) were T1, 1 (14.3%) was T2 and 4 (57.1%) were T4a. One patient (14.3%) was N0, 4 (57.1%) were N2/2b, 1 (14.3%) was N3. Three (42.9%) of patients had confirmed recurrence. All patients were alive at last follow-up. Five (71.4%) tumors originated from the base of tongue (BOT) and 2 (28.6%) originated from the tonsils.

### Survival stratified by tumor risk features

**Fig. 1:**
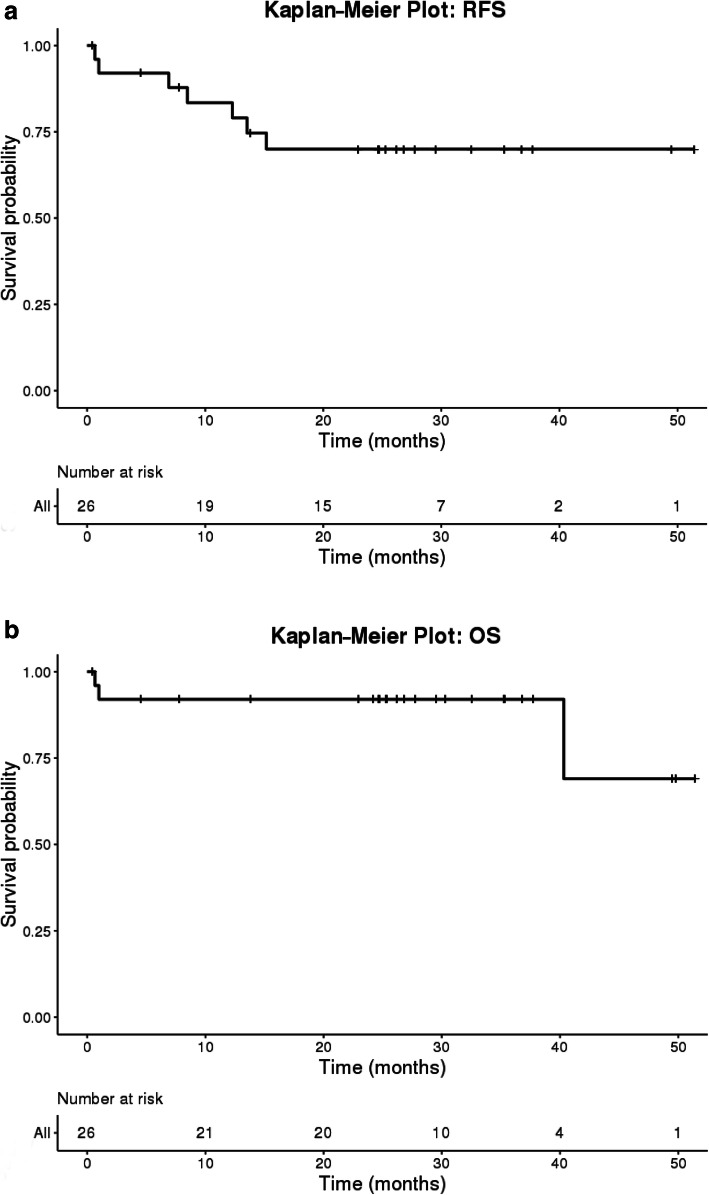
**a** Kaplan Meier Curve for relapse-free survival for the total study sample. **b** Kaplan Meier Curve for overall survival for the total study sample

**Fig. 2:**
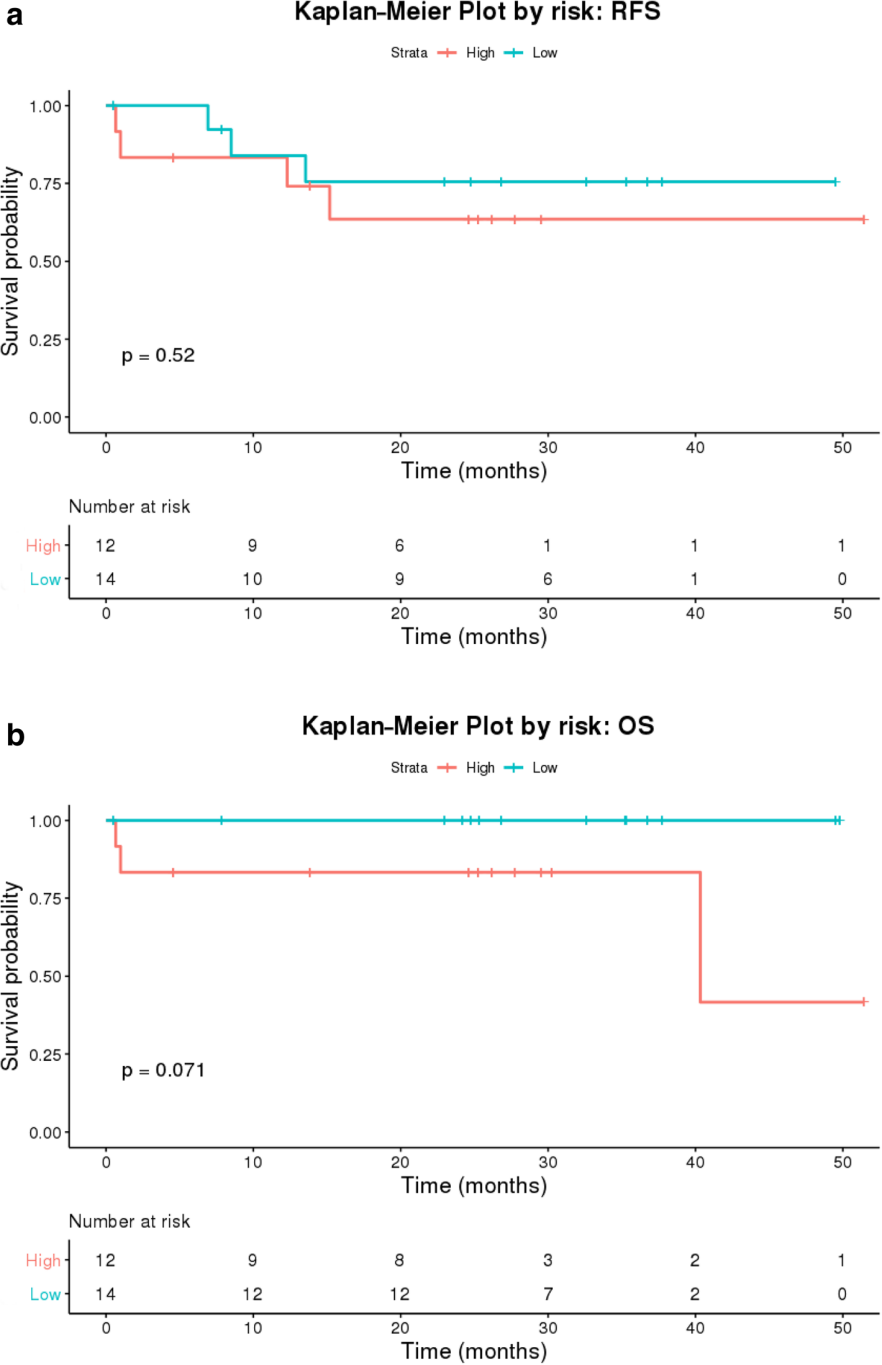
**a** Kaplan-Meier Curves for relapse-free survival for high-risk (red) versus lowrisk (blue) patients in our study sample. **b** Kaplan Meier Curves for overall survival for high-risk (red) versus lowrisk (blue) patients in our study sample

Kaplan-Meier curves for RFS and OS for the entire cohort of 26 patients are included in Fig. 1a and b, respectively. As expected, prognosis of these patients was good with over 90% OS at 3 years. Three-year RFS was 73%. RFS for patients with high-risk features was 67% while those with low- or intermediate-risk features had a 3-year RFS of 79% (Fig. 2a). Importantly, this translated into a 100% 3-year OS for low or intermediate-risk patients compared to 83% in those with high risk features (Fig. 2b). Although these findings were not statistically significant likely due to small sample size, it does suggest that pathological high-risk features in HPV-positive disease are prognostically similar to those with HPV-negative disease. Understanding those at high risk for relapse after surgical resection therefore is important for risk stratification and consideration of alternative treatment modalities.

### Recurrence and survival by biomarker analysis

**Fig. 3:**
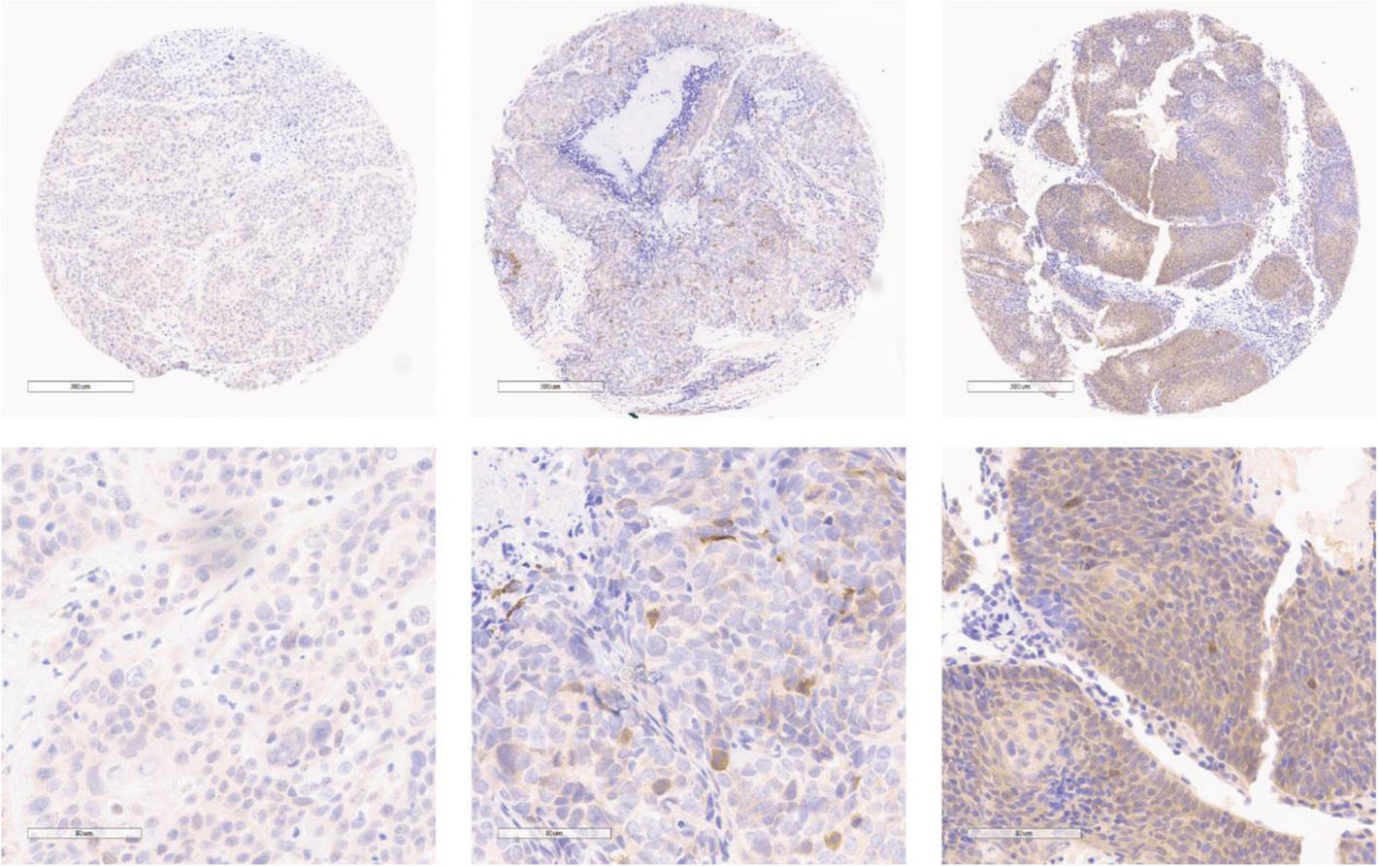
Representative pAMPK immunostaining of a patient’s tumor specimen with low intensity staining (low magnification in top left image and high magnification in bottom left image), medium intensity staining (low magnification in top middle image and high magnification in bottom middle image), and high intensity staining (low magnification in top right image and high magnification in bottom right image)

Molecular biomarker analysis was assessed by immunohistochemistry. Although 26 patients were selected for tissue microarray construction, only 24 patients had quality tissue staining for analysis. Protein expression was scored based on staining intensity and percentage cells that stained positive in each pathology specimen and was correlated with RFS and OS. Figure 3 illustrates representative pAMPK immunostaining in the TMA representing sample patients with low, intermediate and high expression. Interestingly, although not statistically significant, increased pAMPK expression trended towards statistical significance with improved RFS with a hazard ratio of 0.004 and *p* = 0.093 (Table 3). pAMPK expression was not correlated with OS with a hazard ratio of 1.1 (*p* = 0.99). The other molecular markers, including ERCC1, 4EBP1, mTOR, PI3K, PTEN, AKT, and S6 all had hazard ratios very close to 1 for both RFS and OS without statistical significance. There was no discernible correlation between pAMPK expression and surgical margin status, ECE, PNI, or LVI (Table 4). There was also no correlation between expression of any of the other molecular markers and surgical margin status, ECE, PNI, or LVI.

**Table 3:** Correlation between biomarker protein activity and RFS and OS

Risk Factor	RFS HR	*P*-Value	OS HR	*P*-Value
pAMPK	0.004	0.093	1.14	0.999
ERCC1	1.001	0.954	1.043	0.361
p4EBP1	1.000	0.982	1.010	0.508
pmTOR	1.023	0.181	1.042	0.209
PI3K	0.980	0.091	0.990	0.401
PTEN	0.993	0.116	1.004	0.744
pAkt	0.982	0.103	0.992	0.568
pS6	1.002	0.769	0.761	0.998

**Table 4:** Correlation between pAMPK activity and positive margin status, ECE, PNI, and LVI

Outcome	Odds Ratio	*P*-value
Positive Margin Status	1.2	0.11
PNI	1.39	0.11
LVI	1.14	0.26
ECE	1.94	0.36
High Risk	0.9	0.79
Low Risk	1.08	0.44

Molecular biomarker analysis was also assessed using TCGA gene expression data. Only 7 P16 positive patients had recurrence data recorded. RFS was assessed between individuals with increased (≥ median) and decreased (< median) gene expression (Supplemental Figure 1). Though not statistically significant, patients with increased AMPK gene expression had greater RFS as compared to patients with lower AMPK gene expression (*p* = 0.19) (Supplemental Figure 1A). This was similar to the correlation between superior RFS and increased protein expression of AMPK in our cohort. Patients with increased PI3K gene expression had increased RFS (*p* = 0.13) and patients with increased AKT1 gene expression trended toward reduced RFS (*p* = 0.073) (Supplemental Figure 1B,C). RFS curves were also plotted for ERCC1, S6 (RPS6), PTEN, AKT2, AKT3, mTOR, EIF4EP1 (4E-BP1).

## Discussion

Patients with HPV-associated OPSCC often experience significant morbidity with treatment, and in most circumstances treatment includes multiple different modalities. Primary treatment often involves surgery or radiation therapy for localized early stage disease, and surgery followed by adjuvant therapy (radiation therapy if intermediate risk features such as LVI, PNI, or T3/T4 or N2/N3 disease; concurrent chemoradiation for high risk features such as positive surgical margins or ECE) versus primary concurrent chemoradiation for locally advanced disease. Although testing for HPV positivity provides prognostic information, there are insufficient data at the present moment to alter therapy based on HPV status [3]. The most recent 8th edition of the AJCC Staging Manual has incorporated a separate staging schema for HPV+ oropharyngeal squamous cell carcinoma (OPSCC) as compared to HPV-negative OPSCC, however management recommendations have not been altered despite the separate staging for HPV+ disease [26, 27].

Given the improved prognosis, HPV-associated HNSCC has been an attractive target to study de-escalation treatment strategies. However, it should be kept in mind that HPV+ patients are a heterogeneous group, with 75–80% of patients doing very well while the other 20–25% continuing to have a poor prognosis despite aggressive therapy. Accurate and reliable tools have yet to be identified to help differentiate at the molecular level, and at the time of diagnosis, which patients will do well and which ones will do poorly and more importantly, which subset of patients may benefit from less therapy modalities. The ability to risk stratify patients in this way would be very useful as patients known to do better could then potentially be trialed successfully on de-escalation strategies, only subjecting the highest risk patients to intensive multimodality therapy. Randomized trials were recently published comparing cetuximab and RT to cisplatin and RT in HPV positive oropharyngeal cancer and demonstrating inferiority of the cetuximab and RT arm [13, 17]. Perhaps these trials would have benefited from better risk stratification.

We conducted an analysis to try to identify molecular markers within the tumor in HPV-associated head and neck cancer that might help better risk stratify patients and predict recurrence and survival. We analyzed tumor samples from 24 patients and using IHC studied expression of select molecular markers known to be involved in the pathogenesis of HPV+ HNSCC including ERCC1, 4EBP1, AMPK, AKT, S6, PI3K, mTOR, and PTEN. None of these molecular markers portended better outcomes with statistical significance. However, increased pAMPK activation within the tumor was associated with improved RFS with a trend towards statistical significance. Though results were not significant, using the TCGA, there was also a trend that patients with increased AMPK gene expression had longer RFS.

This study was limited by a small sample size. Because our sample size was small, it was difficult to detect meaningful correlations between pAMPK activation and expression of other biomarkers as they relate to recurrence and survival outcomes. It was also difficult to perform adequate subset analyses due to limited sample size, forcing one larger analysis. Further studies into the potential correlation between pAMPK and prognosis are warranted and should be conducted and powered with sample sizes large enough to detect a meaningful difference. Another limitation of this study was that we only looked at a small sample of biomarkers, so there may be others that we didn’t evaluate in this study that may have significance in risk stratification. Finally, this study only looked at a cohort of patients that underwent surgery as their primary treatment modality, so conclusions cannot be expanded to patients undergoing radiotherapy as their primary modality of treatment. It would be interesting to do a similar biomarker analysis in the future of these non-surgical patients to compare and contrast with our surgical cohort.

## Conclusion

Increased pAMPK protein and gene expression was associated with improved RFS with a trend toward statistical significance in this study. This data suggests that high pAMPK activation may portend a better prognosis and therefore patients with HPV-associated OPSCC with high-pAMPK expression in their tumors may be more suitable candidates for de-escalation trials. However, these findings require validation in larger studies.

## Supplementary information

**Additional file 1: Supplemental Figure 1.** Kaplan-Meier plots for RFS in patients with P16+ HNSCC with regards to gene expression.

## Data Availability

The datasets created by the IHC analysis of our TMA that were analyzed to create the final results published in this manuscript can be obtained from the corresponding author on reasonable request. The transcriptome data obtained from TCGA can be accessed via the TCGA website. [https://www.cancer.gov/about-nci/organization/ccg/research/structural-genomics/tcga].

## Abbreviations

BOT: Base of tongue
ECE: Extracapsular extension
HNSCC: Head and neck squamous cell carcinoma
HPV: Human papillomavirus
HR: Hazard ratio
IHC: Immunohistochemistry
LVI: Lymphovascular invasion
OPSCC: Oropharyngeal squamous cell carcinomas
OS: Overall survival
PFS: Progression-free survival
PNI: Perineural invasion
RFS: Relpase-free survival
RT: Radiation therapy
TCGA: The Cancer Genome Atlas
TMA: Tissue microarray
TORS: Transoral robotic surgery
