# Supplementary material for: Determining the molecular landscape and impact on prognosis in HPV-associated head and neck cancer
Source: Cancers Head Neck. 2020 Sep 9;5:11. doi: 10.1186/s41199-020-00058-2 (PMC7487583; doi:10.1186/s41199-020-00058-2)
Supplement: Supplementary file 1 — Additional file 1: Supplemental Figure 1. Kaplan-Meier plots for RFS in patients with P16+ HNSCC with regards to gene expression. [file 41199_2020_58_MOESM1_ESM.docx]

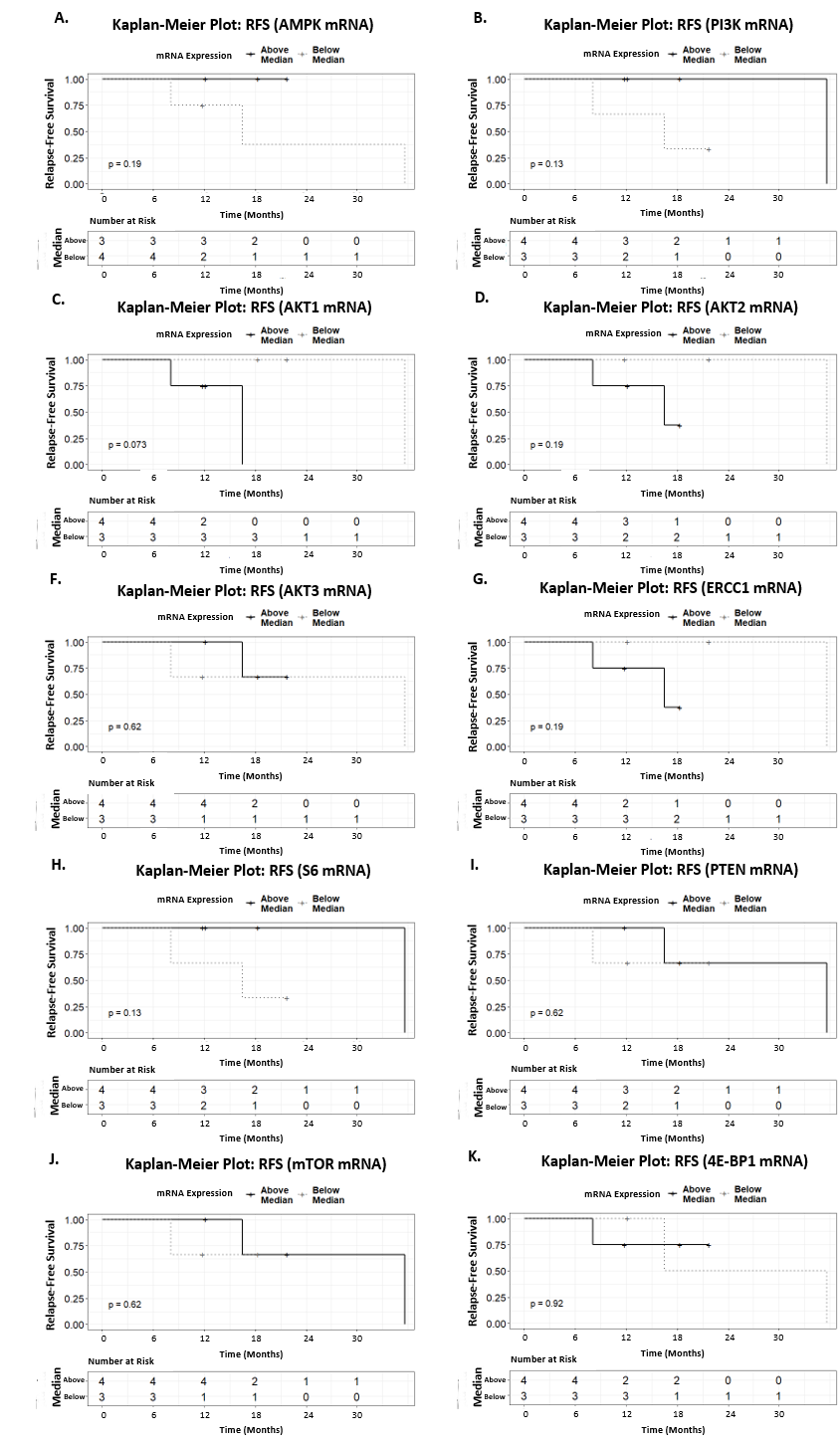
***SUPPLEMENTAL FIGURE 1***

Kaplan-Meier plots for RFS in patients with P16+ HNSCC with regards to gene expression.
